# Slowly progressive dementia caused by MAPT R406W mutations: longitudinal report on a new kindred and systematic review

**DOI:** 10.1186/s13195-017-0330-2

**Published:** 2018-01-09

**Authors:** Emil Ygland, Danielle van Westen, Elisabet Englund, Rosa Rademakers, Zbigniew K. Wszolek, Karin Nilsson, Christer Nilsson, Maria Landqvist Waldö, Irina Alafuzoff, Oskar Hansson, Lars Gustafson, Andreas Puschmann

**Affiliations:** 1 Lund University, Skåne University Hospital, Department of Clinical Sciences Lund, Neurology, Getingevägen 4, 221 85 Lund, Sweden; 2 Lund University, Skåne University Hospital, Department of Clinical Sciences Lund, Diagnostic Radiology, Getingevägen 4, 221 85 Lund, Sweden; 3 Lund University, Skåne University Hospital, Department of Clinical Sciences Lund, Oncology and Pathology, Sölvegatan 23, 221 85 Lund, Sweden; 4 Department of Neuroscience, Mayo Clinic, 4500 San Pablo Road, Jacksonville, FL 32224 USA; 5 Department of Neurology, Mayo Clinic, 4500 San Pablo Road, Jacksonville, FL 32224 USA; 6 Lund University, Skåne University Hospital/Ängelholm Hospital, Department of Clinical Sciences Lund, Memory Clinic, Västersjögatan 10, 262 82 Ängelholm, Sweden; 7 Department of Immunology, Genetics and Pathology, Clinical and Experimental Pathology, Uppsala University, Rudbecklaboratoriet, 75185 Uppsala, Sweden; 8 Lund University, Department of Clinical Sciences Malmö, Clinical Memory Research Unit, Lund, Sweden; 9 Memory Clinic, Skåne University Hospital, 20502 Malmö, Sweden

## Abstract

**Background:**

The *MAPT* c.1216C > T (p.Arg406Trp; R406W) mutation is a known cause of frontotemporal dementia with Parkinsonism linked to chromosome 17 tau with Alzheimer’s disease-like clinical features.

**Methods:**

We compiled clinical data from a new Swedish kindred with R406W mutation. Seven family members were followed longitudinally for up to 22 years. Radiological examinations were performed in six family members and neuropathological examinations in three. We systematically reviewed the literature and compiled clinical, radiological, and neuropathological data on 63 previously described R406W heterozygotes and 3 homozygotes.

**Results:**

For all cases combined, the median age of onset was 56 years and the median disease duration was 13 years. Memory impairment was the most frequent symptom, behavioral disturbance and language impairment were less common, and Parkinsonism was rare. Disease progression was most often slow. The most frequent clinical diagnosis was Alzheimer’s disease. R406W homozygotes had an earlier age at onset and a higher frequency of behavioral symptoms and Parkinsonism than heterozygotes. In the new Swedish kindred, a consistent imaging finding was ventromedial temporal lobe atrophy, which was evident also in early disease stages as a widening of the collateral sulcus with ensuing atrophy of the parahippocampal gyrus. Unlike previously published R406W carriers, all three autopsied patients from the novel family showed neuropathological similarities with progressive supranuclear palsy, with predominant four-repeat (exon 10+) tau isoform (4R) tauopathy and neurofibrillary tangles accentuated in the basal-medial temporal lobe. Amyloid-β pathology was absent.

**Conclusions:**

Dominance of 4R over three-repeat (exon 10−) tau isoforms contrasts with earlier reports of R406W patients and was not sufficiently explained by the presence of H1/H2 haplotypes in two of the autopsied patients. R406W patients often show a long course of disease with marked memory deficits. Both our neuropathological results and our imaging findings revealed that the ventromedial temporal lobes were extensively affected in the disease. We suggest that this area may represent the point of origin of tau deposition in this disease with relatively isolated tauopathy.

**Electronic supplementary material:**

The online version of this article (doi:10.1186/s13195-017-0330-2) contains supplementary material, which is available to authorized users.

## Background

Tauopathies constitute a group of neurodegenerative disorders that include Alzheimer’s disease (AD), progressive supranuclear palsy (PSP), Pick’s disease, and others. These diseases are characterized by cerebral accumulation of the microtubule-associated protein tau [1]. Tau is composed of three- or four-microtubule binding domains, depending on alternative splicing of exon 10, leading to the three-repeat (exon 10−) tau isoform (3R) and the four-repeat (exon 10+) tau isoform (4R). Alternative inserts near the N-terminus result in a total of six tau isoforms expressed under physiological circumstances [2]. Mutations in the gene encoding tau, *MAPT*, can lead to different clinical disorders and neuropathologies, which have been found to depend on the mutation’s location within the gene [2]. Mutations in *MAPT* exons 9 and 11 and most mutations in exons 12 and 13 were associated with combined 3R and 4R tau pathology. Several mutations in exon 10 and all of the 5′ following intronic mutations inhibit alternative exon 10 splicing, leading to predominant 4R tau deposition in neurons as well as glial cells. Other *MAPT* mutations, including *MAPT* c.1216C > T (p.Arg406Trp; R406W) in exon 13, however, did not affect the ratio of 3R to 4R tau isoforms in laboratory models, but they caused aggregations of all six forms of tau, more closely resembling the situation in AD [2]. Clinical symptoms in patients with *MAPT* mutations are generally heterogeneous and designated as frontotemporal dementia with Parkinsonism linked to chromosome 17 (FTDP-17_MAPT_). Patients with R406W develop clinical features of both AD and FTDP-17 [3] and have brain deposition of tau as neurofibrillary tangles (NFTs), whereas amyloid-β (Aβ) pathology is only rarely present [4–6]. This makes FTDP-17_R406W_ an ideal candidate for studying human tau-only pathology.

In this study, we identified R406W in a large Swedish family with slowly progressive dementia. We present longitudinal radiological and clinical data derived from this novel kindred, including the neuropathology of three patients who died after various disease durations. We also systematically reviewed previously described R406W patients, aiming for a firmer determination of the typical clinical features of FTDP-17_R406W_. However, in contrast to previous results, all three neuropathologically examined members of the family in the present study showed PSP-like neuropathology with clear predominance of 4R over 3R tau.

## Methods

### Description of a new kindred

In 1987, the kindred’s proband (III-2) approached one of the authors (LG) because several family members had developed dementia. Family members were contacted after genealogical research in publicly available databases or through the proband, and symptomatic patients were enrolled. Seven affected family members (the proband and patients III-3, III-4, III-5, III-6, IV-9, and IV-16) were followed longitudinally for up to 22 years and were repeatedly examined by a specialist in memory disorders or neurology (LG, CN, or AP). Clinical data, including medical history, neurological and neuropsychiatric assessments, cerebrospinal fluid (CSF) analyses, and apolipoprotein E genotype were collected and compiled. For the other affected members, clinical data as provided by the family were collected.

### Genetic analyses

Blood was collected from a total of 14 family members, and DNA was extracted using standardized methods. Four affected family members (III-2, III-3, III-6, and IV-9) were examined by exome sequencing. Other samples were analyzed with TaqMan genotyping assays (Applied Biosystems, Foster City, CA, USA).

### Imaging

Data on radiological examinations of affected family members performed during the course of the study and at admission were compiled and included computed tomography (CT) and magnetic resonance imaging results. Images were reevaluated in this research study by a neuroradiologist (DvW). Medial temporal atrophy was visually rated using the Scheltens (visual) rating scale [7]. White matter hyperintensities, a common manifestation of small vessel disease, was quantified using the Fazekas scale [8].

### Neuropathology

Autopsy was performed in three family members. Individual III-1 had died in the 1980s of colorectal cancer, and only four small tissue blocks of brain tissue were still available. In individuals III-3 and III-6, full-scale neuropathological examination was performed (by EE) [9]. These brains were assessed macroscopically for regional atrophy and for vascular or other focal pathology, then cut into coronal whole-brain sections as well as into small samples comprising frontal, parietal, temporal, and occipital lobe cortices and white matter, central brain nuclei, limbic structures, brainstem, and cerebellum. After dehydration and paraffin-embedding, the blocks were sectioned at 5- and 4-μm thickness (large and small sections, respectively). Sections from all patients were then stained with conventional stains such as H&E and Luxol fast blue and counterstained with cresyl violet, stains for detection of protein deposits (modified Gallyas silver) and for Aβ (Congo red), as well as immunohistochemical stains targeting hyperphosphorylated tau (AT8; Dako, Glostrup, Denmark), tau isoforms 3R and 4R (anti-tau 3-repeat isoform RD3, 8E6/C11; EMD Millipore, Billerica, MA, USA; and anti-4R-tau, catalogue number TIP-4RT-P01; Cosmo Bio, Tokyo, Japan), α-synuclein (Zymed Laboratories, South San Francisco, CA, USA), and TAR DNA-binding protein (pTDP-43; Cosmo Bio). The tau sections were examined independently by two neuropathologists (EE and IA).

### Systematic review

A systematic review of the R406W literature was performed by searching the PubMed database (latest search date 24 February 2017). Search terms used were “tau”, “MAPT” or “Microtubule associated protein tau” in all possible combinations with “R406W”, “Arg406Trp”, “406”, “1216”, “1216C > T” and “1216C-T”. We included only peer-reviewed publications written in English. Reference lists of the retrieved studies were searched. Case reports and patient series regarding humans with R406W including clinical, radiological, or pathological data were selected for review by reviewing titles and abstracts, as well as, when there was uncertainty, full text. When there were several publications on the same kindred, individual information could be linked to the corresponding family member using data from pedigrees, clinical milestones, or personal communication with the authors. Individuals with no additional data than mutation status were excluded. When particular variables were not reported in a publication, this was marked in the resulting tables. Only descriptive nonparametric statistics were used.

## Results

### Description of a new Swedish kindred

**Fig. 1:**
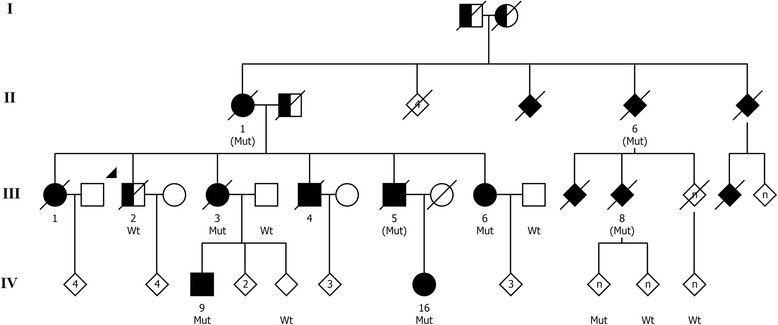
Pedigree of a novel Swedish family with MAPT R406W mutation. *Circles* denote females, *squares* represent males, and *diamonds* indicate that sex was disguised; a *diagonal line* indicates a deceased family member; sibling order was changed, and in some branches, “n” indicates that the number of siblings is not specified, both to protect the family’s confidentiality. *Solid black symbol* indicates individuals with dementia with age at onset before 71 years; *half-filled symbols* indicate dementia with a later age at onset or that clinical information was ambiguous. *Mut* R406W mutation detected by genetic testing, *(Mut)* Obligate R406W carrier, *Wt* Wild type (i.e., R406W mutation excluded by genetic testing). Additional individuals were tested genetically (not shown)

Exome sequencing revealed an R406W mutation in patients III-3, III-6, and IV-9. This mutation was subsequently identified in all other affected family members for whom DNA was available, except the proband (III-2) who had developed dementia during the course of this study but in whom the mutation was excluded. The proband III-2 had onset of memory impairment at the age of 75, which was later than in the other affected family members (Table 1). He was diagnosed with AD 2 years later and did not develop marked behavioral problems or Parkinsonism and was therefore considered to have sporadic AD and was excluded from the study. All other family members who did not carry the mutation were clinically unaffected. Four affected family members for whom no DNA was available were obligate mutation carriers (Fig. 1).

**Table 1:** Clinical data of patients with MAPT R406W mutation in a novel Swedish family

Identifier	R406W	Sex	AoO, years	DD	Clinical diagnosis	APOE	Early or presenting symptoms	Visuospatial	Type of memory impairment	Language impairment	Other cognitive or behavioral symptoms	LoI, psychiatric symptoms	Parkinsonism, other neurological findings
II-1	(Mut)	F	70	9D	Dementia NOS	N/A	N/A	N/A	N/A	N/A	Aggression and wandering behavior	N/A	N/A
III-1	N/A	F	57	2(d)	None	N/A	N/A	N/A	N/A	N/A	Social withdrawal^a^	N/A	None
III-3	Mut	F	53	27D	AD	ε3/ε3	Prosopagnosia^a^	N/A	Episodic	Reduced output, echolalia (26)	Shallow emotions (7), slight disinhibition, confabulation	LoI (8), anxiety, paranoid tendencies	Rigidity and bradykinesia (25), unexplained falls, dyskinesias in lower jaw
III-4	N/A	M	58	17D	AD	ε4/ε4	Memory impairment	Impaired	Episodic and semantic	Apraxia of speech	Disinhibition, aggression and sexual suggestions (9), DV, wandering behavior, confabulation	Severe LoI	None
III-5	(Mut)	M	63	14D	N/A	N/A	N/A	N/A	N/A	N/A	Dyscalculia, dysfunctional at household chores, mild behavioral symptoms (11) wandering behavior (11–14)	Severe LoI, anxiety (14)	None
III-6	Mut	F	50	26D	AD	ε3/ε3	Dyscalculia, social withdrawal, lack of initiative	Mild Q, impaired (24)	Learning difficulties	Reduced spontaneous output	Emotional lability, DV (1), mood changes, perseverations, and confabulation (6), disinhibition, aggression and rude language (18), wandering behavior (24)	LoI, anxiety	Bradykinesia, mild rigidity and dysdiadochokinesia (24), slight tremor^a^, dyspraxia of left hand, dysphagia^a^
IV-9	Mut	M	51	12(a)	MCI	N/A	Irritability^a^, visuospatial difficulties^a^, short-term memory loss	N/A	Short-term memory loss	Dyslexia and dysnomia (5)	Confabulation (5), irritability	Mild LoI, anxiety, emotional lability (5)	None
IV-16	Mut	F	52	6(a)	None	N/A	Anxiety, memory impairment	Q (3)	Episodic (3)	No	Disinhibition	Mild LoI	None

*Mut* R406W heterozygote, *(Mut)* Obligate carrier of R406W, *AoO* Age of (symptom) onset, *DD* disease duration, D indicates disease duration until death, (d) indicates death of an unrelated cause (cancer), and (a) alive, *APOE* Apolipoprotein E genotype, *NOS* Not otherwise specified, *AD* Alzheimer disease, *MCI* Mild cognitive impairment, *N/A* Not available, *LoI* Loss of insight, *DV* Déjà vu-like experiences (i.e. spontaneously recognizing an unknown place or person), *Q* qualitative impairment

Numbers in parentheses indicate disease duration at which particular symptom was reported

^a^Self-reported or reported by relatives

Individual clinical information from the other members of this family (those with the R406W mutation) is compiled in Table 1. Median age of onset was 55 (IQR 51.25–61.75) years, and median disease duration, excluding patient III-1 because her cause of death (colorectal cancer) was considered unrelated to the disease, was 14 (IQR 9–26) years. Memory impairment was a marked symptom in all R406W carriers. Behavioral symptoms were present in all these family members but were mostly predominant in later disease stages and remained mild in several individuals. Other symptoms in this family included confabulation, déjà vu, substance abuse, impaired visuospatial functioning, and wandering behavior. Disease progression was slow (Table 1), and individuals III-3, III-4, III-5, and III-6 did not move to a nursing home until 20, 11, 14, and 24 years after symptom onset, respectively. These four family members also retained the ability to play musical instruments up to 22 years after symptom onset. According to family history, individuals I-1 and I-2 both developed dementia with an escape-prone behavior and had been cared for at a community center for the elderly. Individual II-1 had slowly progressing memory deficits.

Patient III-6 was treated with cholinesterase inhibitors (ChEIs) for 17 years. Treatment effect was uncertain, although further clinical deterioration was documented after ChEI discontinuation. Combination with memantine was discontinued a few weeks after initiation, possibly owing to side effects. Individuals IV-9 and IV-16 have recently begun ChEI treatment.

### Imaging

**Fig. 2:**
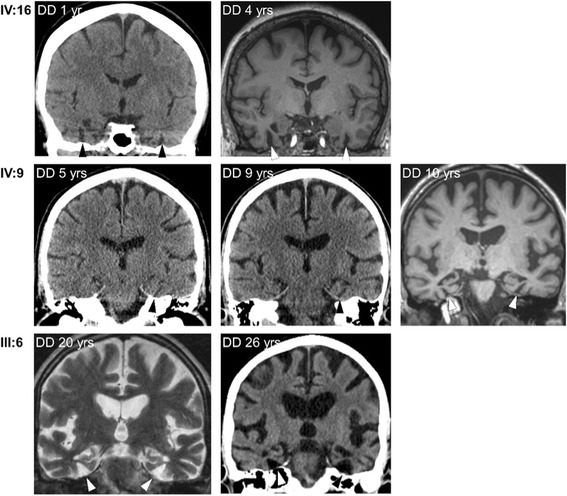
Radiological progression of MAPT R406W-related neurodegeneration. Longitudinal radiological examinations showed increasing ventromedial temporal lobe atrophy with widening of the collateral sulcus (*arrowheads*) as a characteristic and early sign. Hippocampal atrophy was also seen but was only relatively mild to moderate or became obvious only later in the disease course. *DD* Duration of disease at examination

Radiological examinations were performed in six family members, with longitudinal data available from three (Fig. 2). Median disease duration at examination was 6 (IQR 2–20) years in our 6 novel cases compared with 6 (IQR 1–12.25) years for the 23 reviewed cases (Table 2). A pattern was observed in the ventromedial temporal lobe (VMTL) in five family members, characterized by atrophy of the parahippocampal gyrus (PHG) with marked widening of the collateral sulcus (CS). The pattern was observed shortly after symptom onset and progressed over time (Fig. 2). Hippocampal atrophy was present in four family members but less pronounced than PHG atrophy. Thus the temporal atrophy in these patients differed from the typical pattern in AD, where medial temporal lobe atrophy primarily involves the hippocampus with widening of the choroid fissure. In addition, there was general atrophy that increased over time and may be related to both age and disease duration. Individuals IV-9 and III-1 had L > R asymmetric lateral temporal lobe atrophy reminiscent of semantic dementia (SD).

**Table 2:** Radiological findings in carriers of MAPT R406W

	ID	Sex	Modality (n)	DD, years	Atrophy pattern	Temporal lobe atrophy	Hippocampus	Parahippocampal gyrus	Collateral sulcus	Symmetry	Vascular pathology	Quality
Present study	III-1	F	(1) CT	2	T	M 2^a^				L > R	0^b^	–^c^
	III-3	F	(1) CT	28	General	2^a^		Advanced atrophy		Yes	2^b^	–^c^
	III-5	M	(1) CT	6	General	M 2^a^			Widened	Yes	0^b^ + 6-mm lacunar infarction	–^c^
	III-6	F	(1) MRI	20	General, T	M	++ (L > R)	Advanced atrophy	Widened	Yes	0^b^	+
			(1) CT	26	General P, T	M 3^a^		Advanced atrophy	Widened P	Yes	0^b^	++
	IV-16	F	(1) CT	1	Slight T	Slight M	No		Widened	Yes	0^b^	–^c^
			(1) MRI	4	T, P	M P 1^a^			Widened P	Yes	0^b^	++
	IV-9	M	(2) CT	5, 9	General, T	General		Atrophy P	Widened P	L > R	1^b^	
			(2) MRI	10, 10	General, T, F Parietal	General 1^a^		Atrophy P	Widened P	L > R	1^b^	+++
van Swieten et al. [19]	1	F	(1) MRI	4	Mild FT	N/R	N/R	N/R	N/R	L > R	N/R	N/R
	2	M	(1) MRI	5	Mild FT	N/R	N/R	N/R	N/R	L > R	N/R	N/R
Passant et al. [42]	III:1	F	(2) MRI	1–4	T	M and A	Atrophy	N/R	N/R	Yes	N/R	N/R
	III:2	M	(2) MRI	1–4	T	M and A	Atrophy	N/R	N/R	Yes	N/R	N/R
	III:3	M	(2) MRI	1–4	T	M and A	Atrophy	N/R	N/R	Yes	N/R	N/R
	III:4	M	(2) MRI	1	T	M and A	Atrophy	N/R	N/R	Yes	N/R	N/R
Lindquist et al. [4]	III/1	F	(1) CT	N/R	Central	N/R	N/R	N/R	N/R	N/R	N/R	N/R
	III/3	F	(1) CT	N/R	T	N/R	N/R	N/R	N/R	Yes	N/R	N/R
	IV/2	F	(1) MRI	N/R	T	Superficial M	N/R	N/R	N/R	N/R	N/R	N/R
Ikeuchi et al. [43]	P3367-1	M	(1) MRI	N/R	T	M only	Atrophy	Atrophy	N/R	Yes	N/R	N/R
	P3367-2	M	(1) MRI	N/R	T	M	N/R	N/R	N/R	Yes	N/R	N/R
	P3367-3	F	(1) MRI	N/R	T	M	N/R	N/R	N/R	Yes	N/R	N/R
	P3048-1	F	(1) MRI	15	T	M	N/R	N/R	N/R	Yes	N/R	N/R
	P3048-2	F	(1) MRI	N/R	T	M	N/R	N/R	N/R	Yes	N/R	N/R
	P4550-1	F	(1) MRI	9	T	M	N/R	N/R	N/R	Yes	N/R	N/R
Hirschbichler et al. [3]	1	F	(1) MRI	0–1	Normal	N/R	Asymmetric	N/R	N/R	Hippocampal asymmetry	N/R	N/R
Reed et al. [10]	IV-2	M	(1) MRI	0	Normal	N/R	N/R	N/R	N/R	N/R	N/R	N/R
Komatsu et al. [44]	1	M	(1) MRI	1	Mild FT	N/R	Atrophy	N/R	N/R	N/R	N/R	N/R
Tolboom et al. [12]	1	M	(1) MRI	7	Pronounced T	M and A	Atrophy	N/R	N/R	Yes	N/R	N/R
	2	F	(1) MRI	N/R	T	M	Atrophy	N/R	N/R	N/R	N/R	N/R
Ishida et al. [6]	1	M	(1) MRI	N/R	FT	N/R	N/R	N/R	N/R	N/R	N/R	N/R
			(1) CT	N/R	T	N/R	N/R	N/R	N/R	Yes	N/R	N/R
Wood et al. [17]	1	F	MRI	12	T	N/R	N/R	N/R	N/R	R > L	N/R	N/R
	2	F	MRI (>1)	13	T, P	N/R	N/R	N/R	N/R	R > L	N/R	N/R

*DD* Disease duration at examination, *P* Progressive, *T* Temporal, *M* Medial, *A* Anterior/ventral, *FT* Frontotemporal, *MRI* Magnetic resonance imaging, *CT* Computed tomography, *N/R* Not reported

^a^Medial temporal atrophy assessed with Scheltens scale (0–4)

^b^Assessed with Fazekas scale (0–3)

^c^Reduced quality due to thick slices or artefacts

Individuals IV-9, IV-16, and III-6 of this study have been examined by ^18^F-AV-1451 tau-positron emission tomography (PET) in another study revealing tracer uptake in frontotemporal areas and the hippocampus [9].

### Neuropathology

Autopsy of patient III-3, who died after 27 years of disease (brain weight 940 g), macroscopically showed atrophy in the frontal lobes and more severe atrophy bitemporally, especially pronounced in the PHG, accompanied by a marked dilation of the IIIrd and IVth ventricles, as a sign of atrophy within the region of central nuclei and the brainstem, respectively. The hippocampus exhibited severe atrophy, with a remaining size of 4 × 2 mm in the midhippocampal region, coronal plane. Microscopically, there was severe cortical degeneration with neuronal loss and gliosis in the VMTL, including the entorhinal region, where it was most marked in the PHG, creating a markedly widened CS. The amygdala was degenerated, whereas within the hippocampus, the overall severe cell loss was accentuated in Cornu Ammonis (CA)1. There was marked neuronal loss and gliosis of basal ganglia, most pronounced in the globus pallidus. The insular cortex, the thalamus, and the subthalamic nucleus harbored significant tau pathology. Brainstem nuclei were markedly degenerated with cell loss and depigmentation, especially the locus coeruleus and substantia nigra.

Immunoreactivity against hyperphosphorylated tau was intensive in all the areas examined where there was neuronal loss, and there were abundant silver-positive NFTs (Table 3). There was marked white matter degeneration also in the temporal lobe, with widespread tau-positive axonal fragments. In this individual, TDP-43 immunoreactivity was seen, appearing as large cytoplasmic granular inclusions in neurons; fewer but otherwise similar inclusions were present in glial cells in the gray matter and in the white matter.

**Table 3:** Neuropathological features of MAPT R406W patients

Kindred	Patient (DD)	Neuronal tau	Glial tau	Ultrastructure of tau filaments	Tau biochemistry/isoforms	Amyloid	α-Synuclein	Interpretation
Midwest American/FTD004 family [10, 31, 32, 34]	1 (30)	NFT, NT, globose tangles	Occasional astrocytes (tangle-like inclusions)	PHF	Six fractions; both wild-type and mutated tau in deposits	None		PSP
Dutch family [5, 19, 31, 33, 45]	1 (13)	NFT, some pretangles, dystrophic neurites, NT, a few PB	Occasional glial cells	PHF and SF	3R and 4R isoforms; both wild-type and mutated tau in deposits, 4R < 3R	DP (low density), no congophilic plaques, no angiopathy	None	
	2 (13)	NFT, NT, diffuse/amorphous cytoplasmic tau deposits	None	PHF as in AD, and SF (minority of filaments)	3R and 4R isoforms; six fractions; both wild-type and mutated tau in deposits, ghost tangles	NP (moderate density)		
Japanese family 1 [46]	1	NFT, NT	Oligodendroglial coiled bodies (only Gallyas-Braak staining reported)	N/A	Both wild-type and mutated tau in deposits; widespread ghost tangles	None	None	Features of PSP/CBD/tangle-only dementia
U.S. family 2 [35]	1	NFT, NT, no PB	None	N/A	3R and 4R isoforms	None	No LB	NFT dementia
Danish family [4]	1	NFT, NT, no PB	Occasional glial cells (fibrillary inclusions)	N/A	3R and 4R isoforms; 4R < 3R	NP (CERAD A); many DP	None	
Japanese family 2 [6]	1	NFT, NT, PB-like inclusions (or globose tangles)	Cytoplasmic immunoreactivity in glia	N/A	4R < 3R isoforms; 3R-positive ghost cells	NP (CERAD C); many DP		
U.S. family 3 (ZKW, personal communication)	1 (29)	NFT, NT		N/A	3R and 4R isoforms	NP		AD
U.K. patient [17]	1	NFT, PB	Astrocytic plaques	N/R	N/R	N/R	N/R	N/R
Swedish family (present report)	III:1 (2)	NFT, NT, neurites	Tufted astrocytes	N/A	4R > 3R	None	None	PSP-like dementia (minimal material)
	III:3 (27)	NFT, NT, neurites	Tufted astrocytes, glial plaques	N/A	4R > 3R	None	None	PSP-like dementia
	III:6 (26)	NFT, NT, neurites	Tufted astrocytes	N/A	4R > 3R	None	Minimal (focal in CA2)	PSP-like dementia

*AD* Alzheimer’s disease, *3R tau* Three-repeat (exon 10−) tau isoform, *4R tau* Four-repeat (exon 10+) tau isoform, *CA* Cornu Ammonis area, *CERAD* Consortium to Establish a Registry for Alzheimer’s Disease, *DD* Duration of disease, *PHF* Paired helical filaments, *SF* Straight filaments, *NFT* Neurofibrillary tangles, *NT* Neutrophil threads, *PB* Pick bodies, *NP* Neuritic plaques, *DP* Diffuse plaques, *Six fractions* All six insoluble tau fractions detected, *CBD* Corticobasal dementia, *LB* Lewy bodies

The neuropathology of patient III-6, who died after 26 years of disease (brain weight 1085 g), revealed atrophy in the frontal and temporal lobes, more markedly in the latter, as well as dilation of the third and fourth ventricles and atrophy of the brainstem, similar to the macroscopic findings of patient III-3. Microscopically, there was neuronal loss and gliosis in a distribution pattern similar to that of patient III-3, with the majority of pathological changes seen in the basal temporal lobes, including the hippocampus, the central nuclei, and the brainstem nuclei. Also in this patient, there was involvement of cerebral white matter. Tau immunoreactivity was marked owing to NFTs in cortical neurons, the thalamus, PHG, and hippocampi (especially in the dentate fascia), and there were some tufted astrocytes. In the white matter, there were tau-positive depositions in neurites, axonal fragments, and some glial inclusions. The tau-immunoreactive changes were most pronounced in the temporal lobes, clearly evident in the frontal lobes and basal ganglia, less pronounced in the parietal and occipital lobes, and only sparsely in the cerebellum. There was focal α-synuclein-positive pathology with Lewy bodies and Lewy neurites, regionally confined to the hippocampal CA2, where there also was pyramidal cell loss.

Patient III-1 died of colon adenocarcinoma 2 years after onset of memory problems. There were no metastases to the brain. Autopsy was performed in the 1980s. The limited tissue blocks available for reassessment in the present study did not allow a comparison with the areas of maximal macroscopic atrophy on imaging, but they exhibited mild neuronal degeneration in sections representing the midbrain and neocortex (specific area not known). These areas stained densely positive for tau, as did the subjacent white matter.

**Fig. 3:**
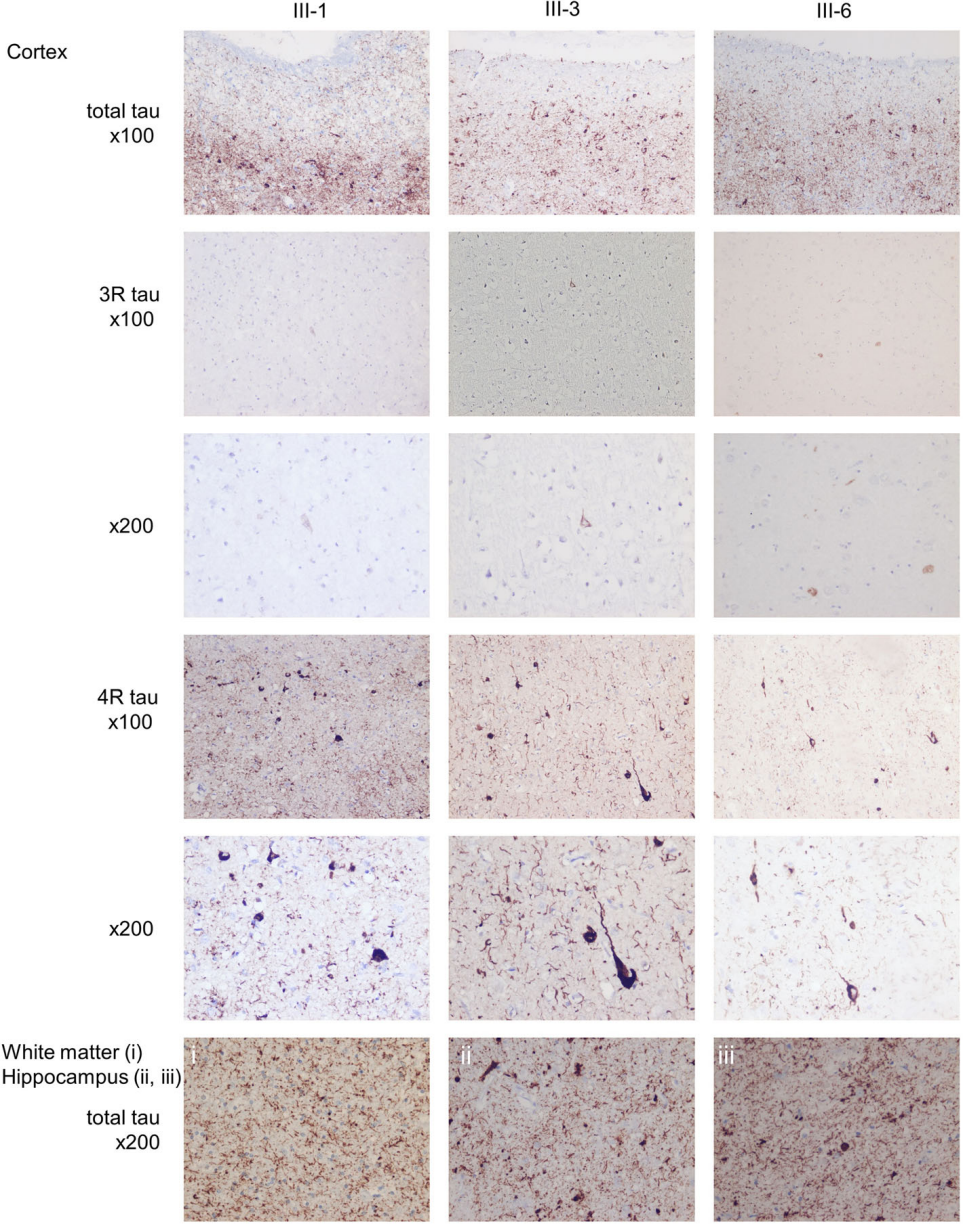
Neuropathology. Photomicrographs of cortical, white matter, and hippocampal sections from three family members. Individuals, immunohistochemistry, and enlargement are as indicated. All three patients had abundant tau pathology in the cortex and white matter, with a marked predominance of four-repeat (exon 10+) tau isoform immunoreactivity. *3R tau* Three-repeat (exon 10−) tau isoform. *4R* *tau* Four-repeat (exon 10+) tau isoform

All three patients assessed neuropathologically had abundant 4R pathology in cells and dystrophic neurites, as well as much less intense and less widespread 3R immunohistochemical staining positivities, which were confined to a few neurons (Fig. 3 and Additional file 1). No brain Aβ was detected. There were many globose NFTs in the cortex, and in patients III-3 and III-6, globose NFTs were also noted in the hippocampus, as well as many tau-positive axonal fragments in the white matter, but there was a less prominent appearance of tufted astrocytes and fewer coiled bodies.

Among the autopsied family members, both III-3 and III-6 carried the H1/H2 tau haplotype. No DNA was available for the third autopsied individual, III-1. Patient IV-9 also showed H1/H2 (Additional file 2).

### Systematic review

Clinical data on 66 R406W patients were compiled from the literature (Table 4, and Additional files 3 and 4 for individual clinical data and cerebrospinal fluid examinations). The reports were published between 1997 and 2017, and methods of ascertainment, patient evaluation, and reporting varied. Median age of onset was 55.5 (IQR 51.25–59.75) years, and median disease duration reported at last examination or death was 13 (IQR 11–21) years. Onset of memory impairment was reported as early in 59% of the reviewed cases. Onset of visuospatial impairment was in the first years of disease for two patients [10, 11] and reported as gradual in four additional patients [11]. Behavioral symptoms developed in 42.4%, but Parkinsonism developed in only 16.7% of reviewed cases. These symptoms were present at disease onset in only 22.7% and 4.5% of the reviewed cases, respectively. Prosopagnosia was reported to have occurred 7 and 24 years after symptom onset in two reviewed cases [10, 12]. Reported R406W homozygotes had much earlier symptom onset and earlier and more pronounced behavioral symptoms and Parkinsonism; one homozygous patient also had paranoid delusions, hyperreligiosity, and compulsive behavior [13, 14].

**Table 4:** Systematic review of descriptions of MAPT R406W patients

References	No. of subjects	Sex	AoO^a^, years	Dur^a^, years	Diagnosis^b^	APOE	Early memory loss (*n*)	Early BS (*n*)	Visuospatial impairment (*n*)	Language impairment (*n*)	BS (*n*)	Psychiatric symptoms (*n*)	Parkinsonism (*n*)	Other (*n*)
[10]	10	7 M, 3 F	54	25	N/R	N/R	7	1	1	1	Aggression 1, Poor judgment and drifting 1	Alcoholism 2	BK, rigidity^c^, action T and mild rest T (arms) 1	No vertical gaze, hypometric saccades, PR 1
[19, 33, 45]	5	1 M, 4 F	57	13	N/R	ε3/ε4 1	3	3	“Mostly intact” 3	Para, pers, flu, mutism 2	N/R	N/R	N/R	N/R
[11]	6^d^	N/R	58	11	EOAD 4, MCI-EOAD 1	ε3/ε3 3, ε3/ε4 2	4	1	5	5	Withdrawal and adapting difficulties 1, Dis 1	Depr and anxiety 1, emotional shallowness 2	Rigidity, cogwheeling 4, T 3, BK 1, gait disturbance 1, total Parkinsonism 4	Dysphagia 1, PR 1
[42, 47]	5	3 M, 2 F	56	23	N/R	N/R	5	4	Normal	Word comprehension 3, stereotypies 3, flu 3, mutism 1	Dietary change 4, dis 2, stereotypies 2, childish 2	Restlessness 3, depr 1, anxiety 1, suspiciousness 2, emotional shallowness 4	No	N/R
[4]	3	0 M, 3 F	65	N/R	AD 2, MCI 1	ε3/ε2 1, ε3/ε3 1, ε3/ε4 1	3	N/R	N/R	N/R	2	N/R	N/R	N/R
[14, 48]	8^ff^	5 M, 2 F	^e^	^e^	AD 1	N/R	N/R	1^f^	N/R	Anomia 2^f^, para 1, comp, flu phonemic and semantic errors impaired word finding, poor spelling and aphasia 1^e^	Apathy 1^f^	N/R	1^f^	N/R
[3]	1	0 M, 1 F	56	N/R	N/R	N/R	1	1	1	N/R	Dis and apathy	N/R	Rest T (R), postural T (bilat), mild BK (L < R), mild rigidity (L < R), and mild dystonia	N/R
[49]	1	1 M, 0 F	50	N/R	N/R	ε3/ε3	1	N/R	N/R	N/R	N/R	N/R	N/R	N/R
[44]	1	1 M, 0 F	66	N/R	N/R	N/R	1	N/R	N/R	N/R	N/R	N/R	N/R	No
[35]	1	0 M, 1 F	54	13	Dementia NOS	ε3/ε3	N/R	N/R	N/R	N/R	N/R	N/R	N/R	N/R
[43, 50, 51]	6	2 M, 4 F	52.5	N/R	EOAD 2, familial AD 1	ε3/ε2 2, ε3/ε3 2, ε3/ε4 2	6	1	N/R	N/R	Loss of interest 1, dis 2, mild 1	Disorientation 1	No 6	N/R
[46]	1	1 M, 0 F	47	6	Schizophrenia, primary degenerative dementia	ε3/ε3	N/R	No	N/R	N/R	Self-neglect	Pdel diminished appetite and severe confusion	N/R	N/R
[6]	1	1 M, 0 F	59	14	AD	N/R	1	No	N/R	Sensory aphasia	Violent, angry, aimless walking, stereotypic	Apraxia, delusional mistakes	No	N/R
[12]	2	1 M, 1 F	56.5	N/R	MCI 1, AD 1	N/R	2	N/R	N/R	N/R	N/R	N/R	N/R	N/R
[52]	6	N/R	57.5	11,5	AD 2, dementia NOS 1	N/R	6	N/R	N/R	5	5	N/R	N/R	N/R
[13]	5^f^	N/R	60–70^e^	N/R	AD 2	ε3/ε3 1^f^	N/R	1^f^	Prosopagnosia 1^f^	Word finding, comprehension and speech output 1^f^	Dietary change, argumentative, and dis 1^f^	Depr^g^, Pdel, hyperreligiosity, compulsory routines, hoarding & Irritability 1^f^	Masked faces and mild BK 1^f^, total Parkinsonism: 4^f^	Slow saccades and square wave jerks^f^
[17]	3	1 M, 2 F	54	13	N/R	N/R	N/R	2	Prosopagnosia 2	Dysnomia 2, repetitive 1	Dietary change 2, stereotypies 1	N/R	No	N/R
[53]	1	1 M, 0 F	38	26	BvFTD	N/R	N/R	N/R	N/R	N/R	N/R	Obsessive, hoarding	N/R	N/R
Summary	66^d,fff^	25 M, 23 F	55 (IQR 51.25–59.75)	13 (IQR 11–21)	AD 17, any 23	Any ε4 5	39	13^h^	6	20^h^	26^h^	13	9^h^	3

*AoO* Median age of onset of individuals reported, *Dur* Duration of disease, *BS* Behavioral symptoms, *AD* Alzheimer’s disease, *EO* Early onset, *MCI* Mild cognitive impairment, *Dementia NOS* Dementia not otherwise specified, *BvFTD* Behavioral variant frontotemporal dementia, *APOE* Apolipoprotein E genotype, *Depr* Depression, *Dis* Disinhibition, *ADL* Activities of daily living, *Para* Paraphasias, *Pdel* Paranoid delusions, *Pers* Perseverations, *PR* Primitive reflexes (e.g., snout, grasp, rooting or palmomental), *Flu* Reduced verbal fluency or output, *Comp* Compulsive repetition of words, *BK* Bradykinesia, *T* Tremor

^a^Reported as median of individual cases

^b^Clinical diagnosis/diagnosis prior to genetic testing

^c^Possible pharmacological cause (neuroleptics)

^d^Including one R406W carrier with subjective memory deficit

^e^Unsatisfactorily reported to be added to summary

^f^Each superscript letter f indicates one confirmed MAPT R406W homozygote

^g^Reported prior to onset of cognitive impairment

^h^Summary of status of MAPT R406W heterozygotes but not homozygotes

Radiological and neuropathological data of previously described cases were compiled (Tables 2 and 3). Stabilized clinical progression and positive fludeoxyglucose-PET measurement was reported in one patient after memantine treatment [4]. Neuropathological examination was performed in nine reviewed cases. Four had tau-only pathology, but five showed co-occurring Aβ pathology, including several cases with observed concomitant positive immunohistochemistry for Aβ and ubiquitin (Table 3).

### CSF examinations

Results of CSF examinations were available for three members of the novel Swedish kindred and six reported cases in the literature and showed a trend for increased levels of CSF tau and phosphorylated tau in about half of R406W patients, but normal levels of CSF Aβ_1–42_ (Additional file 4).

## Discussion

We present a novel Swedish R406W kindred with markedly slowly progressive dementia, as well as a systematic review of previous reports on FTDP-17_R406W_ patients. The clinical phenotype of patients with this mutation consists predominantly of memory loss and varying or gradual development of frontotemporal dementia (FTD)-like cognitive dysfunction. Radiological examinations of FTDP-17_R406W_ patients reveal progressing atrophy that starts in the VMTL and spreads to adjacent regions over time. Neuropathology shows a pattern of regional degeneration and accumulation of NFTs. Although previously reported individuals with this mutation had both 3R and 4R tau pathology, all three autopsied members of the present family had a PSP-like pathology, with a regional spread of degeneration and with marked predominance of 4R over 3R, but without some of the other PSP-characteristic traits.

### Limitations

The major limitation of this study is the small size of our cohort and the low quantity of verified R406W cases in the literature. The review might be influenced by publication or selection biases as well as differences in reporting and grading of symptoms between authors. A common problem with neurogenetic diseases is the rarity of specific point mutations [15]. This is an important issue because larger cohorts will probably be needed to explain the observed and not yet fully understood clinical heterogeneity of patients with *MAPT* mutations.

### Clinical features

Although there is large intra- and interfamilial clinical variability in all FTDP-17_MAPT_ [2], R406W patients often first develop memory impairment. Parkinsonism occurred rarely at end-stage disease in the novel Swedish family, and many patients in this and other families with FTDP-17_R406W_ received an initial clinical diagnosis of AD. Disease progression was remarkably slow (Tables 1 and 2), and age of onset is later than for patients with most other *MAPT* mutations [2]. In the novel Swedish family, déjà vu and confabulation, previously described in dementia cases with predominant temporal lobe atrophy [16], developed in 50% of cases. As the disease progressed, more symptoms typically associated with FTD developed, such as disinhibition, wandering behavior, and aggression. More severe disease was present in all of the more recently described homozygous R406W patients, suggesting a dose-dependent and direct effect of R406W mutated tau [13, 14].

### Radiological features

Atrophy in our novel R406W cases was initially seen in the VMTL, with atrophy of the PHG and secondary widening of the CS as a consistent, early, and progressive pattern. Throughout the disease course of these patients, VMTL atrophy remained more pronounced than hippocampal atrophy, which was observed only late. Thus, the pattern of atrophy in our R406W cases differed from the typical AD pattern. Any assessment of diagnostic or prognostic value of this finding was considered beyond the scope of this work, and confirmation is needed. We suggest, however, that relatively isolated PHG atrophy and CS widening should raise suspicion of FTDP-17_MAPT_ and especially FTDP-17_R406W_. Because radiologists may tend to describe either medial or lateral temporal lobe atrophy, and because neither atrophy of the PHG nor associated widening of the CS is incorporated in the Scheltens (visual) rating scale [7], there may be underreporting of more predominant VMTL atrophy, and/or localized changes affecting only CS or PHG, in other tauopathies.

FTDP-17_R406W_ disease can sometimes manifest with asymmetric temporal lobe atrophy [17], and left (dominant) lateral temporal lobe atrophy has rarely been reported in FTDP-17_R406W_ patients [2, 18, 19]. Clinical SD symptoms in addition to FTD phenotype, however, have been more commonly reported in FTDP-17 patients with other *MAPT* mutations [20]. Temporal lobe atrophy with left-sided dominance, as in SD, was found radiologically in patients III-1 and IV-9. The unrelated and early death of patient III-1 prevented detailed characterization of clinical symptoms, but patient IV-9 had dysnomia and misunderstood or forgot instructions despite relatively short disease duration. This supports the possibility of an SD-similar phenotype in FTDP-17_R406W_.

This is the first study to report mainly 4R tau pathology in FTDP-17_R406W_ patients, which contrasts with previous findings [2]. It has been suggested that the pattern of temporal lobe atrophy differs, depending on the location of the *MAPT* mutation, with regard to the effect on exon 10 alternative splicing [21]. Medial temporal lobe atrophy is found more often in patients with IVS10 + 3, IVS10 + 16, p.N279K, and p.S305N mutations, which affect exon 10 splicing and lead to expression of only 4R tau. Lateral temporal lobe atrophy was reported in p.P301L and p.V337M cases, where both 3R and 4R tau are expressed [2, 21].

### Neuropathological features

In the novel Swedish family, individuals III-1, III-3, and III-6 had tau depositions in cortical neurons and in white matter, which were most pronounced in the temporal lobe. In particular, the widening of the CS, as seen with imaging, prevailed as a prominent detail also demonstrated postmortem (Additional file 1). The temporolimbic region was thus particularly affected (when available for neuropathological examination; patient III-3 and III-6), and furthermore, the locus coeruleus had a high tau load and was highly atrophic. These findings are consistent with the clinical similarities between R406W-associated disease and AD. In the hippocampi of patients III-3 and III-6, CA1 neurons were severely affected, similar to both PSP and AD. The severity of the central atrophy exceeded that seen in other tauopathies, except in severe forms of PSP. This unequally spread atrophy might stand for a slow and unique distribution of pathology caused by R406W mutated tau.

Immunohistochemical staining in the novel family showed the presence of both 3R and 4R tau isoforms. However, 4R tau staining was clearly more abundant, and 3R tau was judged to be at nearly physiological levels for age (Fig. 3). The pathological picture in the Swedish family was reminiscent of PSP also in aspects other than 4R predominance: (1) the presence of neuronal NFTs and, albeit scarce, of tufted astrocytes and occasional coiled bodies [22, 23]; (2) prominent tau white matter pathology, and (3) marked involvement of the brainstem and central nuclei. However, the cortical load of tau-positive threads and neurons was more dense and severe than seen in typical PSP [22]. This may hypothetically be an effect of the disease process allowed to develop during an unusually long time. Hereditary PSP is rare, but families with more than one member with pathologically proven PSP have been described, including a family harboring an MAPT p.S285R mutation [24].

Other genetic or environmental factors can probably modify neurodegenerative features in human tauopathies. The H1 *MAPT* haplotype increases PSP risk and has been associated with higher expression of 4R tau, but it is too common in the general population to explain the unusual 4R tau predominance in the present family [25–27] and has previously been shown not to influence the phenotype of FTDP-17 patients with other *MAPT* mutations [28]. A more complex interaction between the R406W mutation and other genetic variability in the H1/H2 clades cannot be excluded. It has also been shown that the pathology of PSP changes with regard to disease duration [29], and in AD, the predominance of either the 4R or 3R isoform of tau pathology similarly depends on disease duration; however, more 4R tau was present in early than in late AD stages [30].

Aggregation of tau and subsequent atrophy in our R406W patients was most pronounced in the VMTL area. The transentorhinal region is also prominently affected by tau pathology with NFT accumulation in early idiopathic AD, and additional findings overlap between AD and FTDP-17_R406W_, including clinical symptoms, expression of all isoforms of tau with laboratory findings of a 3R/4R ratio close to 1, ultrastructural composition of tau in paired helical filaments, with a minority of short filaments [4, 31–35]. Furthermore, R406W mutated tau has been shown to increase its toxicity in the presence of Aβ [36]. The locus coeruleus may be an even earlier starting point for tau pathology in AD [37] and was severely affected by tau pathology in patients III-3 and III-6. Aβ pathology was found in five FTDP-17_R406W_ patients, four of whom had neuritic plaques ([4–6]; ZKW, personal communication). Interestingly, Aβ levels have been shown to be increased to just below the plaque formation threshold in the brains of patients with FTD with *MAPT* mutations [38]. NFT formation in the VMTL is also a hallmark in primary age-related tauopathy (PART), formerly named *tangle-only dementia*. Aβ deposition is believed never to ensue in PART [35], which is presumed to be a more purely age-related NFT pathology [39], but its role as an entity distinct from AD is debated [40, 41]. Despite the many similarities between FTDP-17_R406W_ and AD, our understanding is that FTDP-17_R406W_ tauopathy is more clearly distinct from AD than PART is, owing to the genetic basis of the disease. The above-mentioned similarities might thus imply dual pathogenic mechanisms rather than comorbidity.

## Conclusions

FTDP-17_R406W_ is a distinct disease entity that clinically shares features with AD, but in a novel Swedish family we found it to be associated with PSP-like, predominant 4R tau pathology, most pronounced in the VMTL, and we found a persistent and characteristic imaging pattern of VMTL atrophy. This disease provides an interesting model for the pathomechanisms of tau pathogenesis in humans.

## Additional files

Additional file 1: Figure S1. Additional neuropathology. Additional photomicrographs from three family members. Individuals, immunohistochemistry, and enlargement as indicated. (TIF 11179 kb)

Additional file 2: Table S1. *MAPT* haplotype determination based on exome sequencing data. Individuals, residues, and haplotype as indicated. Note that the proband, individual III-2, had Alzheimer’s disease and was excluded from the study. (PDF 181 kb)

Additional file 3: Table S2. Individual data from previous publications (systematic review). Numbers indicate disease duration at first reported or observed symptom. Data in parentheses are successive or vaguely specified development or data unspecifically described. *Symptoms reported by patient or relative. ^#^Unreliable data owing to possible pharmacological cause (neuroleptics). ^§^Data too imprecisely reported to be included in calculations. *AD* Alzheimer’s disease, *MCI* Mild cognitive impairment, *Dementia NOS* Dementia not otherwise specified, *CSF* Cerebrospinal fluid, *Aβ*_*42*_ Amyloid-β 1–42, *APOE* Apolipoprotein E, *ADL* Activities of daily living. (PDF 161 kb)

Additional file 4: Table S3. Cerebrospinal fluid examinations. Numbers in parentheses indicate disease duration when cerebrospinal fluid was retrieved. *Upward filled triangles* = value above laboratory’s reference value for healthy individuals; *downward filled triangles* = value below laboratory’s reference value for healthy individuals. ^a^Compared with healthy control subjects (157 ± 65 pg/ml, *n* = 24) and patients with AD (555 ± 248 pg/ml, *n* = 31). ^b^Compared with healthy control subjects (29 ± 10 pg/ml, *n* = 21) and patients with AD (86 ± 39 pg/ml, *n* = 31). *N/A* Not assessed. (PDF 304 kb)

## Abbreviations

3R tau: Three-repeat (exon 10−) tau isoform
4R tau: Four-repeat (exon 10+) tau isoform
Aβ: Amyloid-β
AD: Alzheimer’s disease
ADL: Activities of daily living
AoO: Age of onset
APOE: Apolipoprotein E
BK: Bradykinesia
BS: Behavioral symptoms
BvFTD: Behavioral variant frontotemporal dementia
CA: Cornu ammonis
CBD: Corticobasal degeneration
CERAD: Consortium to Establish a Registry for Alzheimer’s Disease
ChEI: Cholinesterase inhibitor
Comp: Compulsive repetition of words
CS: Collateral sulcus
CSF: Cerebrospinal fluid
CT: Computed tomography
DD: Duration of disease
Dementia NOS: Dementia not otherwise specified
Depr: Depression
Dis: Disinhibition
DP: Diffuse plaques
DV: Déjà vu-like experiences (i.e., spontaneously recognizing an unknown place or person)
EO: Early onset
EOAD: Early-onset Alzheimer’s disease
Flu: Reduced verbal fluency or output
FTD: Frontotemporal dementia
FTDP-17: Frontotemporal dementia with Parkinsonism linked to chromosome 17
LB: Lewy bodies
LoI: Loss of insight
MCI: Mild cognitive impairment
MRI: Magnetic resonance imaging
Mut: R406W heterozygote
(Mut): Obligate carrier of R406W
NFT: Neurofibrillary tangle
NP: Neuritic plaques
NT: Neutrophil threads
Para: Paraphasias
PART: Primary age-related tauopathy
PB: Pick bodies
Pdel: Paranoid delusions
Pers: Perseverations
PET: Positron emission tomography
PHF: Paired helical filaments
PHG: Parahippocampal gyrus
PR: Primitive reflexes
PSP: Progressive supranuclear palsy
Q: qualitative impairment
SD: Semantic dementia
SF: Straight filaments
T: Tremor
VMTL: Ventromedial temporal lobe
