# Supplementary figures and images for: Slowly progressive dementia caused by MAPT R406W mutations: longitudinal report on a new kindred and systematic review
Source: Alzheimers Res Ther. 2018 Jan 9;10:2. doi: 10.1186/s13195-017-0330-2 (PMC6389050; doi:10.1186/s13195-017-0330-2)

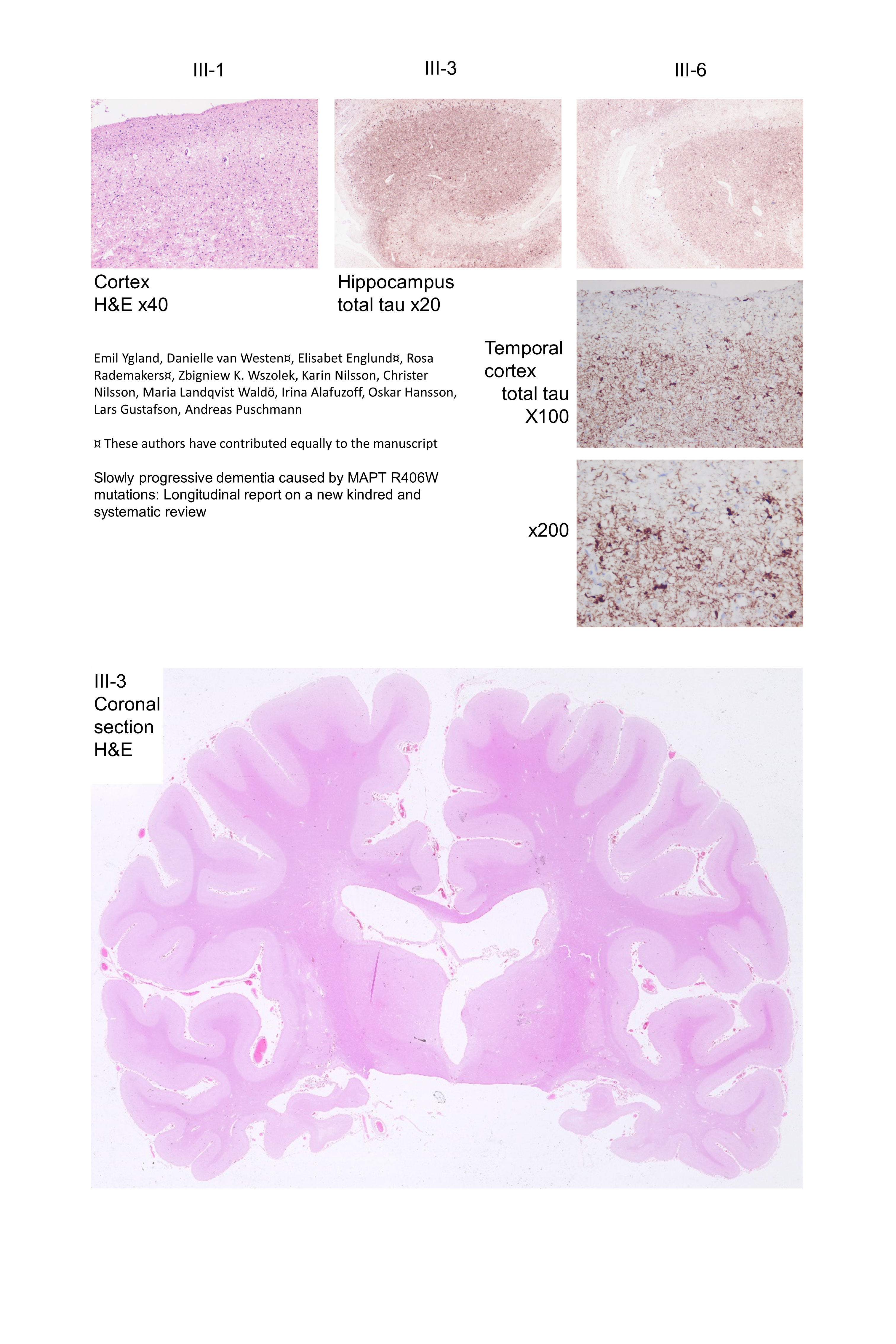

Supplement: Supplementary file 1 — Additional neuropathology. Additional photomicrographs from three family members. Individuals, immunohistochemistry, and enlargement as indicated. (TIF 11179 kb) [file 13195_2017_330_MOESM1_ESM.tif]
