# Supplementary material for: Slowly progressive dementia caused by MAPT R406W mutations: longitudinal report on a new kindred and systematic review
Source: Alzheimers Res Ther. 2018 Jan 9;10:2. doi: 10.1186/s13195-017-0330-2 (PMC6389050; doi:10.1186/s13195-017-0330-2)
Supplement: Supplementary file 2 — MAPT haplotype determination based on exome sequencing data. Individuals, residues, and haplotype as indicated. Note that the proband, individual III-2, had Alzheimer’s disease and was excluded from the study. (PDF 181 kb) [file 13195_2017_330_MOESM2_ESM.pdf]

Slowly progressive dementia caused by MAPT R406W mutations: Longitudinal report on a new kindred and systematic review

Emil Ygland, Danielle van Westen¤, Elisabet Englund¤, Rosa Rademakers¤, Zbigniew K. Wszolek, Karin Nilsson, Christer Nilsson, Maria Landqvist, Irina Alafuzoff, Oskar Hansson, Lars Gustafson, Andreas Puschmann

¤ These authors have contributed equally to the manuscript

Corresponding author: Andreas Puschmann, MD, PhD; affiliation: Lund University, Skåne University Hospital, Department of Clinical Sciences Lund, Neurology; Email: andreas.puschmann@med.lu.se

	**III:2 (proband, no R406W mutation)**	**III:3 (R406W)**	**III:6 (R406W)**	**IV:9 (R406W)**
rs1052551	G/G	G/A	G/A	G/A
rs1052553	A/A	A/G	A/G	A/G
rs17652121	T/T	T/C	T/C	T/C
Haplotype	**H1/H1**	**H1/H2**	**H1/H2**	**H1/H2**
