# Supplementary material for: Slowly progressive dementia caused by MAPT R406W mutations: longitudinal report on a new kindred and systematic review
Source: Alzheimers Res Ther. 2018 Jan 9;10:2. doi: 10.1186/s13195-017-0330-2 (PMC6389050; doi:10.1186/s13195-017-0330-2)
Supplement: Supplementary file 3 — Individual data from previous publications (systematic review). Numbers indicate disease duration at first reported or observed symptom. Data in parentheses are successive or vaguely specified development or data unspecifically described. *Symptoms reported by patient or relative. #Unreliable data owing to possible pharmacological cause (neuroleptics). §Data too imprecisely reported to be included in calculations. AD Alzheimer’s disease, MCI Mild cognitive impairment, Dementia NOS Dementia not otherwise specified, CSF Cerebrospinal fluid, Aβ42 Amyloid-β 1–42, APOE Apolipoprotein E, ADL Activities of daily living. (PDF 161 kb) [file 13195_2017_330_MOESM3_ESM.pdf]

Slowly progressive dementia caused by MAPT R406W mutations: Longitudinal report on a new kindred and systematic review

Emil Ygland, Danielle van Westen¤, Elisabet Englund¤, Rosa Rademakers¤, Zbigniew K. Wszolek, Karin Nilsson, Christer Nilsson, Maria Landqvist Waldö, Irina Alafuzoff, Oskar Hansson, Lars Gustafson, Andreas Puschmann
¤ These authors have contributed equally to the manuscript

Corresponding author: Andreas Puschmann, MD, PhD; affiliation: Lund University, Skåne University Hospital, Department of Clinical Sciences Lund, Neurology; andreas.puschmann@med.lu.se

Authour	Pat name	R406W genotype	Gender	Age of onset	Duration	Death	Duration at nursing home	Clinical diagnosis prior to genetic testing	APOE genotype	Early symtoms	Viso-spatial	Type of memory impairment	Executive function	Language impairment	Behavioral symtoms	Insight	Psychiatric symtoms	Escape prone	Gaze	Parkinsonism	Bradykinetic	Dystonia	Rigidity	Tremor/cogwheeling	Cogwheeling	Primitive reflexes/other	Imbalance	Other diseases	Effect of drug
Present study	1		f	57	2	y	n/a								Social withdrawal														
	2	Heterozygote	f	53	27	y	20	AD	E3/E3	Prosopagnosia?		Confabulation		Ekolalia 26	Emotionally flat (7)		Anxiety, paranoid tendencies				(25)					No			
	3		m	63	17	y	14								Early	Impaired		14											
	4		m	58	17	y	11	AD	E4/E4 enl percys journal 2004	Impaired memory, spatial impairment, do not remember names of new collegues.	Impaired	Learning difficulties		Paraphasias	Disinhibition, confabulation, aggression and sexual suggestions (9)	Impaired		Yes											
	5	Heterozygote	f	50	26	y	24	AD	E3/E3	Dyscalkuli, social withdrawal	Qualitative shortages	Learning difficulties, confabulation 6			Social withdrawal and lack of initiative (0), easily provoked affectively (1), shallow emotions suddenly shifting to aggression & rude language (18)	Impaired		24			Late					Dysphagia, late			
	6	Heterozygote	f	52	3	n	n/a	developped symptoms after genetic testing		Anxiety and memory problems during employment interview, imparied memory	Qualitative shortages	Episodic 3		No	Disinhibition														
	7	Heterozygote	m	51	9	n	n/a	MCI		Short term memory loss		Short term memory loss, confabulation 5			Short tempered 0,Easily provoked affectively 5,														
	8		f	70	9	y	n/a								Aggression, wander of														
[10]	III-9	Heterozygote	m	47	29					Impaired memory, orientation	0	Reduced digit span, severely impaired antegrade memory. Prosapagnosia (24)		Paucicity (24), palaliacy (29)	Aggression (29)				No vertical, hypometric saccades in endstage	Late	Bradykinesia and rigidity (24), action tremor and mild rest tremos in upper extrimity		24#	Action tremor and mild rest tremor in arms		No vertical gaze, hypometric saccades, snout and rooting reflexes (29)			
	I-1	Heterozygote	m			y																							
	II-2	Heterozygote	m	55	21		y			Impaired memory																			
	II-3	Heterozygote	f	45	35		y			Impaired memory																			
	II-4	Heterozygote	m	55						Behavioural					Poor judgement, eccentric, drifting (0)														
	II-5	Heterozygote	m		20		y			Impaired memory																			
	III-3	Heterozygote	m	75						Impaired memory, confusion																			
	IV-1	Heterozygote	f	54						Mildly impaired memory																			
	IV-2	Heterozygote	m	49						Mild antegrade amnesia		Impaired antegrade memory														No focal neurologic deficits			
[19, 33, 45]	III:2	Heterozygote	m	56	13					Impaired memory, initiative loss and restlessness	Mostly intact			Mutism (10)															
	III:3	Heterozygote	f	53	13					Impaired memory, initiative loss and restlessness	Mostly intact			Mutism (8)															
	III:4	Heterozygote	f	57	13				E3/E4	Impaired memory, initiative loss and restlessness	Mostly intact			No (12)															
	II:1	Heterozygote	f	65	17									(naming paraphasias, verbal perseveration and impaired verbal fluency in nearly all patients)															
	II:6	Heterozygote	f	65	11																								
[11]	4	Heterozygote	f	56	23			EOAD	E3/E4	Impaired memory, dyskalculi	(1-12)			Abnormal (17) Mutism (23)	Flat affect (23)					16 severe	(17)		23	Tremor and cogwheeling (23)	23	Snout, palmoomental and grasp (23)		Earlier depression, surgery at >15 years duration	
	6	Heterozygote	n/a	59	11			EOAD, PD	E3/E3	Withdrawal, adapting difficulties	Gradually			Complete aphasia (7)	Withdrawal and adapting difficulties (0), flat affect (5)					(0-5)			(5-11)	Cogwheeling (5-11)	5-11	Dysphagia (5-11)		No (surgery 30 years prior to diagnosis)	1-Methyldopa
	8	Heterozygote	n/a	62	22			EOAD	E3/E3	Impaired memory	Gradually			Aphasia (22)	No					15			Face (22)	Mild tremor (15), cogwheeling (22)	22			Vascular risk	
	13	Heterozygote	n/a	48	6	y		EOAD	E3/E4	Disorientation, impaired speech	(0)			(0)	Disinhibition, inappropriate joking (5)					No								No	
	14	Heterozygote	n/a	58	8			MCI-EOAD	E3/E3	Prosapagnosi	Gradually			Late	No		Depression, anxiety			7				Cogwheeling 7	Very mild, assymetric 7			No	
	15	Heterozygote	n/a	n/a				Subjective memory deficit	E3/E3	Delayed recall					No					No									
[42, 47]	II:1	Heterozygote	f	55	23	y				Impaired episodic memory, personality change, restlessness	Normal	Episodic 0	Slightly impaired (4)	Word comprehension (11-20), Mutism (20)	Personality change 1-4 Disinhibition, Emotionaly shalownes & Dietary change 11-20	Some	Depression 1-4			No								No	
	III:1	Heterozygote	f	62						Impaired episodic memory, personality change, restlessness	Normal	Episodic 0	Slightly impaired (4)	Word comprehension and verbal fluency (1-4), Stereotypic speech (11)	Personality change 1-4, emotionall shallowness, stereotyped behaviour & dietary change 11-20	Some	Anxiety 1-4			No								No	
	III:2	Heterozygote	m	60						Impaired episodic memory, restlessness	Normal	Episodic 0	Slightly impaired (4)	Word comprehension, Stereotypic speech, reduced output/verbal fluency (11)	Personality change, disinhibition, emotionall shallowness, stereotyped and childish behaviour, dietary change, irritability 11-20	Some				No								No	
	III:3	Heterozygote	m	54						Impaired episodic memory, childish behaviour, irritability	Normal	Episodic 0	Slightly impaired (4)	Stereotypic speech and reduced output (11) word comprehension	Personality change, childish behaviour & irritability 1-4, emotionall shallowness & dietary change 11-20	Some	Suspiciousness 1-4			No								No	
	III:4	Heterozygote	m	56						Impaired episodic memory, childish behaviour	Normal	Episodic 0	Slightly impaired (4)		1-4	Some	Suspiciousness 11-20			No								No	
[4]	III-1	Heterozygote	f	65				AD	E3/E3	Impaired memory					Mid-Late														
	III-3	Heterozygote	f	65				AD	E3/E4	Impaired memory					Mid-Late														
	IV-2	Heterozygote	f	52				MCI	E3/E2	Slight prosapagnosia, Impaired memory		Deminished recall			No														Memantine
[14, 48]	V-7	Homozygote	m	39§				FTDP-17		Language impairment, anomia, impaired behaviour, parkinsonism				Anomia, compulsive repetition of words, nonfluent and hesitant speech, phonomic and semantic errors, impaired word finding, poor spelling 0, aphasia (5)	Disturbed (0), Apathy (5)					0	0		0	Rest 0			Yes		

	V-4		f	35§	7§	y																							
	V-5	Homozygote	m	35§	7§	y																							
	V-6		m	35§	7§	y																							
	Brother-a		m	35						Language impairment, anomia				Prominent including paraphasic errors															
	Brother-b	Heterozygote	m	50																									
	Brother-c	Heterozygote	m	58																									
	IV-1	Heterozygote	f	70				AD																					
[3]	1	Heterozygote	f	56	n/a					Parkinsonism, forgetfull*, apathic*, disinhibited	Impaired		Impaired		Disinhibited appearence					Yes (0)	Mild right sided (0-1)	Mild (0-1)	Mild right side (0-1)	Rest in right hand + bilateral posture (0)					
[49]	15625	Heterozygote	m	50					E3/E3	Impaired memory																			
[44]		Heterozygote	m	66						Impaired memory										Normal neurological investigation									
[35]	15	Heterozygote	f	54	13	y		Dementia NOS	E3/E3			AD-like																	
[43, 50, 51]	P3367-2	Heterozygote	m	45				EO-FAD	E3/E4	Impaired memory, Disorientation, loss of interest		Anterograde episodic			Loss of interest		Disorientation			No									
	P3367-1	Heterozygote	m	51				EOAD	E3/E4	Impaired memory		Anterograde episodic								No									
	P3367-3	Heterozygote	f	50					E2/E3	Impaired memory		Anterograde episodic			Mild personality change and disinhibition (17)					No									
	P3048-1	Heterozygote	f	56				FAD	E3/E3	Impaired memory		Anterograde episodic								No									
	P3048-2	Heterozygote	f	54					E3/E3	Impaired memory		Anterograde episodic			Mild disinhibition but relatively preserved frontal executive functioning (13)					No									
	P4550-1	Heterozygote	f	68					E2/E3	Impaired memory		Anterograde episodic			Mild personality change and disinhibition (9)					No									
[46]		Heterozygote	m	47	6	y		Schizophrenia, Primary Degenerative Dementia	E3/E3	Paranoid delusions					Self neglect (5)		Paranoid delusions 0, diminished apetite and severe confusion 5												
[6]	1	Heterozygote	m	59	14	y		AD	N/A	Impaired memory				Sensoral aphasia 8	Violent, angry, aimless walking, stereotypic 8		Apraxia, delusional misstakes 8			No								Tuberculous osteomyelitis, deafness post streptomycin 0, chronic renal failure (14)	
[12]	1	Heterozygote	m	54				MCI (FAD suspected)		Impaired memory		Impaired registration and retention of verbal information, prosapagnosia (7)	Normal 7																
	2	Heterozygote	f	59				AD (FAD suspected)		Impaired memory, impaired ADL		Registration and retention of verbal information	Normal																
[52]	III-1	n/a	n/a	57	13					Impaired memory				Mid-late	Mid-late														
	III-4	n/a	n/a	55	17																								
	IV-1	n/a	n/a	58	7			Dementia NOS																					
	IV-2	n/a	n/a	65	11			AD																					
	IV-11	n/a	n/a	55	12			AD																					
	IV-12	n/a	n/a	60	16																								
[13]	1 Hom	Homozygote	m	33					E3/E3	Dietary change, argumentative	Subjective (1), prosapagnosia (6)			Word finding and comprehension & speech output (6)	Dietary change & argumentative (0), Disinhibition (3)		Depression (presymtomatic), paranoid delusions & hyperreligousity (3), compulsory rutines (5), hoarding & Irritability (6)			Masked faces & mild bradykinesia (7)								Impaired memory, impaired executive functions	
	Parent 1		N/R	60-70§																									
	Parent 2		N/R	50-60§													Y			Y									
	Grandparent 1		N/R	60-70§				AD									Y			Y									
	Great grandparent 1		N/R	60-70§				AD									Y			Y									
[17]	1		f	54	12						Prosapagnosia			Fluent but devoid of content & anomia (12)	Stereotypia, dietary change					No (12)								Affected memory, frontal dysexecutive	
	2		f	51	13									Repititive (10), Dysnomia (13)	Confabulation, dietary change					No (13)								Impaired memory (10)	
	Father of 2		m	before 65§	21						Prosapagnosia																		
[53]	1		m	38	26																								
