# Supplementary material for: Slowly progressive dementia caused by MAPT R406W mutations: longitudinal report on a new kindred and systematic review
Source: Alzheimers Res Ther. 2018 Jan 9;10:2. doi: 10.1186/s13195-017-0330-2 (PMC6389050; doi:10.1186/s13195-017-0330-2)
Supplement: Supplementary file 4 — Cerebrospinal fluid examinations. Numbers in parentheses indicate disease duration when cerebrospinal fluid was retrieved. Upward filled triangles = value above laboratory’s reference value for healthy individuals; downward filled triangles = value below laboratory’s reference value for healthy individuals. aCompared with healthy control subjects (157 ± 65 pg/ml, n = 24) and patients with AD (555 ± 248 pg/ml, n = 31). bCompared with healthy control subjects (29 ± 10 pg/ml, n = 21) and patients with AD (86 ± 39 pg/ml, n = 31). N/A Not assessed. (PDF 304 kb) [file 13195_2017_330_MOESM4_ESM.pdf]

Slowly progressive dementia caused by MAPT R406W mutations: Longitudinal report on a new kindred and systematic review

Emil Ygland, Danielle van Westen¤, Elisabet Englund¤, Rosa Rademakers¤, Zbigniew K. Wszolek, Karin Nilsson, Christer Nilsson, Maria Landqvist Waldö, Irina Alafuzoff, Oskar Hansson, Lars Gustafson, Andreas Puschmann

¤ These authors have contributed equally to the manuscript

Corresponding author: Andreas Puschmann, MD, PhD; affiliation: Lund University, Skåne University Hospital, Department of Clinical Sciences Lund, Neurology; Email: andreas.puschmann@med.lu.se

**Reference**	**Patient ID**	**Total tau** (pg/ml)	**Phosphorylated tau** (pg/ml)	**Amyloid beta** (pg/ml)	**Neurofilament light chain** (pg/ml)	**Glial fibrillary acidic protein** (pg/ml)
Present study	III:3	N/A	Normal (3)	Normal (3)	N/A	N/A
	III:6	**427** ▲ (7) [ref: <260]; 300 (20) [ref: <400]	54 (20) [ref: <80]	860 (20) [ref: >450]	<250 (20) [ref: <750]	N/A
	IV:9	**400** ▲ (5), 396 (9) [ref: <400]	48 (5) [ref: <60]; 49 (5) [ref: <80]	910 (5) [ref: >450]; 957 (9) [ref: >550]	630 (5) [ref: <890]; 400 (9) [ref: <1850]	230 (9) [ref: <1250]
	IV:16	**528** ▲ (3) [ref: <400]	58 (3) [ref: <60]	1010 (3) [ref: >550]	NFL 780 (3) [ref: <890]	500 (3) [ref: <750]
[25]	IV-2	Normal	Normal	Normal	N/A	N/A
[15]	P3367-1	**282** ▲ [ref: [a]]	**80** ▲ [ref: [b]]	N/A	N/A	N/A
	P3367-2	116 [ref: [a]]	**74** ▲ [ref: [b]]	N/A	N/A	N/A
[17]	1	**728** ▲ [ref: <202**]**	**89** ▲ [ref: <49]	**341** ▼ [ref: >490]	N/A	N/A
[44]	1	340 [ref: <356]	51 [ref: <54]	933 [ref: >495]	N/A	N/A
	2	**442** ▲ [ref: <356]	**63** ▲ [ref: <54]	1026 [ref: >495]	N/A	N/A
